# Dynamic microbial changes in exacerbation of chronic obstructive pulmonary disease

**DOI:** 10.3389/fmicb.2024.1507090

**Published:** 2024-12-06

**Authors:** Yong Jun Choi, Hye Jung Park, Chi Young Kim, Aeri Choi, Jae Hwa Cho, Min Kwang Byun

**Affiliations:** Division of Pulmonary and Critical Care Medicine, Department of Internal Medicine, Gangnam Severance Hospital, Yonsei University College of Medicine, Seoul, Republic of Korea

**Keywords:** chronic obstructive pulmonary disease, microbiome, exacerbations, microbial diversity, respiratory health, disease mechanisms

## Abstract

**Background:**

Microbial profiles in patients with chronic obstructive pulmonary disease (COPD) provide insights for predicting, preventing, and treating exacerbations. This study aimed to analyze the impact of microbial diversity and spectrum on COPD exacerbation.

**Methods:**

From November 1, 2018, to May 31, 2023, we prospectively enrolled patients with stable disease (SD) and exacerbation of COPD (ECOPD). Sputum samples were collected for microbiome DNA sequencing, and amplicon sequence variants were analyzed.

**Results:**

We collected sputum samples from 38 patients: 17 samples from patients with SD and samples from patients with ECOPD at two time points—during exacerbation (AE-1: 21 samples) and again during stabilization after 2 weeks of treatment (AE-2: 17 samples). Alpha diversity indices, specifically observed feature count and Fisher’s alpha index, were significantly higher in SD (133.0 [98.0–145.0]; 17.1 [12.7–19.6]) compared to AE-1 (88.0 [72.0–125.0], *p* = 0.025; 10.9 [8.5–16.1], *p* = 0.031). The SD showed significantly higher abundances of *Neisseria* (linear discriminant analysis [LDA] 4.996, adj.*p* = 0.021), *Fusobacterium* (LDA 3.688, adj.*p* = 0.047), and *Peptostreptococcus* (LDA 3.379, adj.*p* = 0.039) at the genus level compared to AE-1. At the species level, *N. perflava* (LDA 5.074, adj.*p* = 0.010) and *H. parainfluenzae* (LDA 4.467, adj. *p* = 0.011) were more abundant in SD. Hub genera in the microbial network included *Haemophilus*, *Granulicatella*, *Neisseria*, *Lactobacillus*, and *Butyrivibrio* in SD and *Streptococcus*, *Gemella*, *Actinomyces*, *Klebsiella*, and *Staphylococcus* in AE-1.

**Conclusion:**

COPD exacerbations are linked to changes in specific strains of normal flora. Maintaining microbial diversity and balance within the microbial network is critical for preventing and managing COPD exacerbations.

## Introduction

Chronic obstructive pulmonary disease (COPD) is a heterogeneous lung condition characterized by chronic respiratory symptoms due to airway and/or alveolar abnormalities, leading to persistent and often progressive airflow obstruction (Celli et al., 2022). COPD exacerbation (ECOPD) is characterized by a rapid onset of worsened respiratory symptoms such as dyspnea, cough, and increased sputum within 14 days (Celli et al., 2021). These exacerbations are commonly linked to local and systemic inflammation, often triggered by infections or airway irritants (Celli et al., 2021). ECOPD not only increases patient mortality but also imposes significant economic burdens (Park et al., 2022). Therefore, developing effective preventive and treatment strategies is crucial (Park et al., 2022).

Emerging evidence indicates that respiratory tract microecological disorders play a role in COPD pathogenesis, particularly through alterations of the airway microbiome, which contributes to neutrophilic inflammation in COPD and ECOPD (Xue et al., 2023; Karakasidis et al., 2023). Additionally, the overall microbiome diversity, rather than specific strains, has been highlighted in ECOPD pathogenesis (Xue et al., 2023; Pragman et al., 2024). A diverse and balanced microbiome may be crucial in maintaining respiratory health and preventing exacerbations. Consequently, novel therapeutic strategies targeting the lung microbiome for managing and treating ECOPD have been suggested (Li et al., 2024; Sin, 2023).

This study aims to investigate alterations in the airway microbiome during ECOPD and assess their correlation with clinical outcomes.

## Materials and methods

### Patients and study design

From November 1, 2018, to May 31, 2023, we conducted a prospective screening of all patients who visited the outpatient clinic of Gangnam Severance Hospital. COPD was defined according to the recommendations of the Global Initiative for Chronic Obstructive Lung Disease (GOLD) 2018 as patients presenting symptoms of COPD or those with risk factors and a post-bronchodilator FEV_1_/FVC ratio of less than 0.7. Patients were included if they met the diagnostic criteria for COPD. The exclusion criteria were as follows: patients with asthma, those unable to provide sputum samples, and those who had already received antibiotic treatment within the previous month. Stable Disease (SD) was defined as the absence of ECOPD episodes in the past year. ECOPD was defined as an acute worsening of respiratory symptoms requiring additional therapy. Sputum samples were collected from patients with SD and ECOPD on their enrollment dates (SD and AE-1, respectively). Additionally, patients with ECOPD provided a second sputum sample (AE-2) 2 weeks after initiating treatment with antibiotics, corticosteroids, or both.

Qualified spontaneous sputum samples, based on epithelial cell counts, were collected within 24 h of enrollment before any systemic therapy (SD and AE-1). We considered samples with epithelial cells <10/LPF in sputum Gram stain grading as uncontaminated by oropharyngeal secretions. AE-2 sputum samples were collected 2 weeks after medical treatment with antibiotics, corticosteroids, or both, either at outpatient clinics or upon hospital admission for ECOPD.

### Library construction and sequencing

DNA was extracted using the DNeasy PowerSoil Pro Kit (Qiagen, Hilden, Germany) following the manufacturer’s protocol. Extracted DNA was quantified using Quant-IT PicoGreen (Invitrogen).

Sequencing libraries were prepared following the PacBio Amplicon Template Preparation and Sequencing protocols, targeting the 27F and 1492R regions. Input gDNA (2 ng) was polymerase chain reaction (PCR)-amplified with 10× LA PCR Buffer II (Mg^2+^-free), 2.5 mM dNTP mix, 2.5 mM MgCl_2_, 500 nM each of forward and reverse PCR primers, and 5 U of TaKaRa LA Taq (Takara, Kusatsu, Japan). The PCR conditions were initial heat activation at 94°C for 5 min, followed by 25 cycles of 30 s at 94°C, 30 s at 53°C, and 90 s at 72°C, with a final extension at 72°C for 5 min. The primer pairs with asymmetric barcoded adapters used for the amplifications were: 27F-F: 5′- AGRGTTYGATYMTGGCTCAG −3′ and 1,492-R: 5′- RGYTACCTTGTTACGACTT -3′. PCR products were purified using SMRTbell cleanup beads, quantified using Quant-IT PicoGreen (Invitrogen), and qualified using the TapeStation D5000 Screen Tape (Agilent Technologies, Waldbronn, Germany). For PacBio Sequel IIe sequencing, 500 ng of pooled amplicon DNA was used for library preparation. A total of 10 μL of the library was prepared using the PacBio SMRTbell prep kit 3.0. SMRTbell templates were annealed using the Sequel II Bind Kit 3.1 and Int Ctrl 3.1. Sequencing was performed using the Sequel II Sequencing Kit 2.0 and SMRT cells 8 M Tray. Data acquisition involved 10-h movies captured for each SMRT cell using the PacBio Sequel IIe (Pacific Biosciences) sequencing platform, conducted by Macrogen (Seoul, Korea). Subsequent steps followed the PacBio Sample Net-Shared Protocol, which is available on the PacBio website.^1^

### Statistical analysis

We conducted a power analysis to evaluate the required sample size. With an effect size of 0.5, an alpha error of 0.05, and a power of 0.80, we set a target of including at least 35 participants.

Categorical variables were presented as frequencies (percentages). Continuous variables were presented as mean ± standard deviation (SD) for normally distributed data and as median (interquartile range [IQR]) for non-normally distributed data. Normality assumptions for continuous variables were confirmed using the Shapiro–Wilk test. Baseline characteristics among three or more distinct patient groups were compared using one-way analysis of variance (ANOVA) or Kruskal–Wallis tests. Tukey’s Honest Significant Difference test served was used post-hoc for ANOVA, while Dunn’s test followed the Kruskal–Wallis test. Fisher’s exact test was used to analyze categorical data.

Analyses were performed using R (R Foundation for Statistical Computing, Vienna, Austria)^2^. Phyloseq, microbiomeMarker, and NetCoMi packages were used for ASV sequencing, linear discriminant analysis (LDA) effect size (LEfSe), and network analyses, respectively. The MaAsLin2 package was used to apply a mixed-effects linear model for paired samples to conduct a within-patient analysis. A Spearman’s correlation plot was constructed using the Corrplot package. Permutational multivariate analysis of variance (PERMANOVA) was performed using the vegan package.

## Results

### Baseline characteristics

Thirty-eight patients were enrolled in the study, with 17 assigned to the SD group and 21 to the AE group (Table 1). Seventeen sputum samples were obtained once from the SD group and 38 samples were obtained twice from the AE group, taken at two different time points. Four AE-2 samples were excluded from longitudinal analysis in AE due to follow-up loss.

**Table 1:** Baseline characteristics of enrolled patients.

Group	AE	SD	*p*-value
	(*N* = 21)	(*N* = 17)	
Age (years)	74.6 ± 7.3	70.4 ± 9.0	0.119
Sex (males)	20 (95.2%)	16 (94.1%)	1.000
Height (cm)	168.0 [161.5;171.3]	168.0 [163.2;171.0]	0.803
Weight (kg)	**59.7 ± 9.6**	**67.3 ± 11.2**	**0.030**
BMI (kg/m^2^)	**21.9 [20.6;23.0]**	**24.0 [22.1;25.3]**	**0.025**
History of antibiotic use	18 (85.7%)	17 (100.0%)	0.308
History of steroid use	19 (90.5%)	17 (100.0%)	0.564
Use of antibiotics	**14 (66.7%)**	**3 (17.6%)**	**0.007**
Use of steroid	**20 (95.2%)**	**2 (11.8%)**	**<0.001**
History of smoking			0.350^†^
Non-current smoker	18 (85.7%)	14 (82.4%)	1.000^‡^
Never smoker	7 (33.3%)	9 (52.9%)	
Ex-smoker	11 (52.4%)	5 (29.4%)	
Current smoker	3 (14.3%)	3 (17.6%)	1.000^‡^
Sputum collection season			0.576
Spring	8 (38.1%)	3 (17.6%)	
Summer	4 (19.0%)	4 (23.5%)	
Autumn	4 (19.0%)	5 (29.4%)	
Winter	5 (23.8%)	5 (29.4%)	
Underlying disease (Charlson comorbidity index)			
Myocardial infarction	2 (9.5%)	1 (5.9%)	1.000
Congestive heart failure	1 (4.8%)	1 (5.9%)	1.000
Peripheral VD	3 (14.3%)	4 (23.5%)	0.757
Central VD	9 (42.9%)	3 (17.6%)	0.190
Dementia	0 (0.0%)	1 (5.9%)	0.915
Rheumatic disease	0 (0.0%)	0 (0.0%)	1.000
Peptic ulcer disease	4 (19.0%)	4 (23.5%)	1.000
DM without complication	3 (14.3%)	2 (11.8%)	1.000
DM with complication	2 (9.5%)	0 (0.0%)	0.564
Renal disease	5 (23.8%)	5 (29.4%)	0.984
Any tumor	5 (23.8%)	2 (11.8%)	0.595
Metastatic tumor	2 (9.5%)	0 (0.0%)	0.564
COPD grade			
1	**0 (0.0%)**	**1 (5.9%)**	**0.003**
2	**5 (23.8%)**	**13 (76.5%)**	
3	**11 (52.4%)**	**3 (17.6%)**	
4	**5 (23.8%)**	**0 (0.0%)**	
Chest x-ray findings			
Pneumonia	6 (33.3%)	2 (22.2%)	0.882
Bronchitis	12 (66.7%)	4 (44.4%)	0.489
Fibrosis	2 (11.1%)	2 (22.2%)	0.848
Emphysema	10 (55.6%)	4 (44.4%)	0.892
Edema	1 (5.6%)	0 (0.0%)	1.000

BMI, body mass index; VD, vascular disease; COPD, chronic obstructive pulmonary disease; DM, diabetes mellitus.

^† The p-value was calculated for comparisons among never, ex-, and current smokers across groups.^

^‡ The p-value was calculated for comparisons between non-current smokers and current smokers across groups.^

The bold values indicate statistically significant results with *P* < 0.05.

Although no significant differences were observed in age, sex, height, history of smoking, sputum collection season, underlying diseases, and initial chest radiographic findings between groups, weight and body mass index (BMI) were significantly lower in the AE than in SD (59.7 ± 9.6 kg vs. 67.3 ± 11.2 kg, *p* = 0.030; and 21.9 kg/m^2^ [IQR 20.6–23.0] vs. 24.0 kg/m2 [22.1–25.3], *p* = 0.025, respectively, Table 1). The AE group showed a higher use of antibiotics (66.7%) compared to the SD group (17.6%), with a *p*-value of 0.007. Similarly, steroid use was more frequent in the AE group (95.2%) than in the SD group (11.8%), with a *p*-value of <0.001.

AE patients had more severe COPD grades, with a higher proportion in grade 3 (52.4%) and grade 4 (23.8%), while SD patients were mostly in grade 2 (76.5%), showing a significant difference in COPD grade distribution (*p* = 0.003).

Additionally, in the analysis for longitudinal assessment within the AE group, there were no significant differences in baseline characteristics between the included and excluded patients (Supplementary Table 1).

Pulmonary function tests revealed that forced expiratory volume in the first second (FEV_1_) was significantly lower in AE (1.1 liters [0.8–1.5] vs. 1.7 liters [1.5–2.1], *p* = 0.001) as was z-score of FEV_1_ by Global Lung Function Initiative-2012 (GLI-2012) reference (−3.3 [−3.8–-2.7] vs. −2.3 [−2.6–−2.0], *p* < 0.001). Forced expiratory flow (FEF, FEF_25–75%_, FEF_25_, FEF_50_, and FEF_75_) and peak expiratory flow were lower in the AE group than in the SD group (Table 2).

**Table 2:** Clinical features of enrolled patients.

Group	AE	SD	*p*-value
	(*N* = 21)	(*N* = 17)	
Laboratory data			
Hemoglobin	13.6 ± 1.9	14.6 ± 1.6	0.087
White blood cells	7.7 [6.6; 8.8]	7.8 [6.7; 9.2]	0.599
Eosinophil	0.1 [0.0; 0.2]	0.2 [0.1; 0.2]	0.217
Platlet	240.8 ± 74.0	245.5 ± 103.4	0.870
Blood urea nitrogen	15.0 [9.1; 18.2]	17.9 [13.6;19.8]	0.158
Creatinine	0.8 [0.7; 1.1]	0.9 [0.9; 1.1]	0.328
AST	28.0 [20.0;32.0]	22.0 [19.0; 30.0]	0.327
ALT	21.0 [16.0;26.0]	16.0 [12.0; 29.5]	0.618
C-reactive protein	2.0 [0.6; 18.3]	1.2 [0.7; 1.8]	0.246
Spirometers			
FVC (liters)	3.2 ± 0.9	3.5 ± 0.7	0.326
FVC (z-score)	−0.5 ± 1.3	−0.0 ± 1.1	0.260
FEV_1_ (liters)	**1.1 [0.8; 1.5]**	**1.7 [1.5; 2.1]**	**0.001**
FEV_1_ (z-score)	**−3.3 [−3.8; −2.7]**	**−2.3 [−2.6; −2.0]**	**<0.001**
FEV_1_/FVC (%)	**33.5 [28.5; 44.5]**	**53.0 [42.0; 60.0]**	**0.002**
FEV_1_/FVC (z-score)	−4.3 ± 0.3	−4.4 ± 0.3	0.222
FEF_25-75%_	**0.2 [0.2; 0.5]**	**0.6 [0.4; 0.8]**	**0.005**
FEF_25_	**0.8 [0.5; 1.7]**	**2.0 [1.7; 3.5]**	**0.001**
FEF_50_	**0.3 [0.2; 0.7]**	**0.8 [0.5; 1.2]**	**0.003**
FEF_75_	0.1 [0.1; 0.2]	0.2 [0.1; 0.3]	0.079
PEF	**3.4 [2.5; 5.1]**	**5.0 [4.3; 5.9]**	**0.010**

AST, aspartate aminotransferase; ALT, alanine aminotransaminase; FVC, forced vital capacity; FEV1, forced expiratory volume in 1 s; FEF, forced expiratory flow; PEF, peak expiratory flow. The bold values indicate statistically significant results with *P* < 0.05.

### Diversity of airway microbiomes in patients with COPD

In 55 sputum samples, 3,470 amplicon sequence variants were obtained, with a median feature sequence frequency of 36,284.

**Figure 1:**
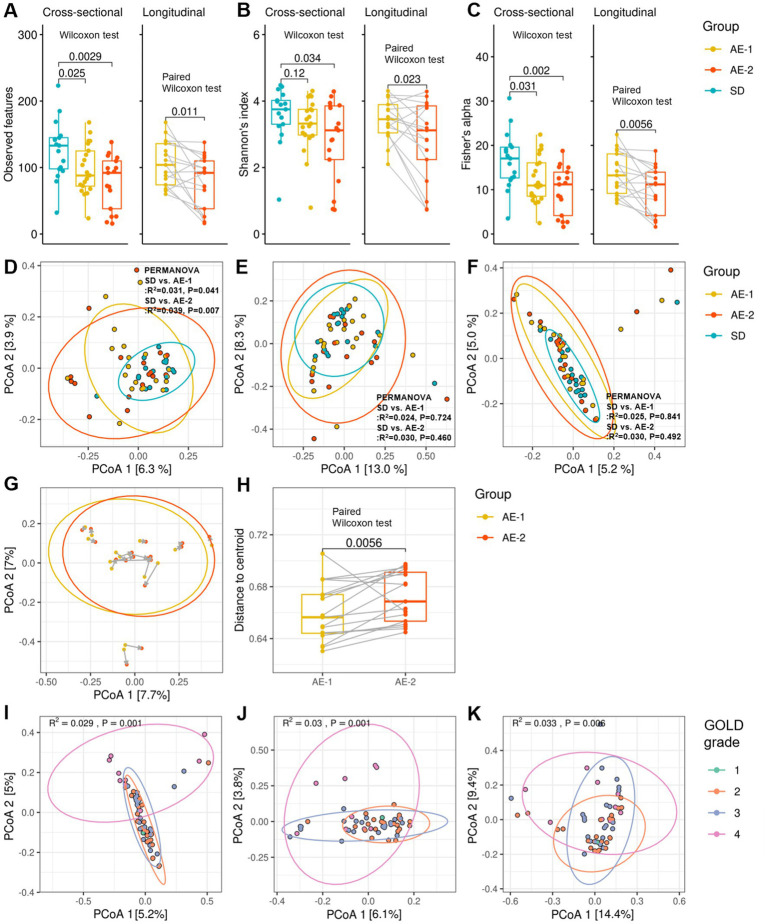
Diversity of airway microbiome in patients with COPD. Panels **(A–C)** display whisker-bar plots comparing alpha diversity across the SD, AE-1, and AE-2 groups, showing observed features, Shannon’s index, and Fisher’s alpha index, respectively. Cross-sectional comparisons were performed using the Wilcoxon test, and longitudinal comparisons were assessed with the paired Wilcoxon test. Panels **(D–F)** present cross-sectional comparisons of beta diversity across the SD, AE-1, and AE-2 groups, showing unweighted UniFrac, weighted UniFrac, and Bray–Curtis dissimilarity, respectively, with *p*-values calculated by PERMANOVA. Panel **(G)** illustrates changes in Bray–Curtis dissimilarity between AE-1 and AE-2 samples, where gray arrows indicate a 2-week interval. Panel **(H)** presents a longitudinal comparison of Bray–Curtis dissimilarity centroid distances, assessed by paired Wilcoxon test. Panels **(I–K)** present cross-sectional comparisons of beta diversity across chronic obstructive pulmonary disease grades 1 to 4.

In alpha diversity, the observed feature count was significantly higher in SD (133.0 [98.0–145.0]) compared to AE-1 (88.0 [72.0–125.0], *p* = 0.036) and AE-2 (92.0 [38.0–110.0], *p* = 0.002; Figure 1A). Shannon’s index was also significantly higher in SD (3.8 [3.3; 4.0]) than in AE-2 (3.1 [2.2; 3.9], *p* = 0.034; Figure 1B). Similarly, Fisher’s alpha index was higher in SD (17.1 [12.7; 19.6]) compared to AE-1 (10.9 [8.5; 16.1], *p* = 0.031) and AE-2 (11.2 [4.2; 14.0], *p* = 0.002; Figure 1C). The under-73 group also showed significantly higher diversity metrics compared to the 73-and-older group, with an observed feature count of 137.3 ± 32.8 vs. 82.9 ± 32.7, Shannon’s index of 3.9 [3.5–4.2] vs. 3.0 [2.8–3.6], and Fisher’s alpha index of 18.2 ± 4.9 vs. 10.4 ± 4.6 (all *p* < 0.001; Supplementary Figure 1A). After adjusting for age through stratified analysis, the SD group showed higher alpha diversity indices compared to the AE-1 group (Supplementary Figure 2A). In contrast, the alpha diversity indices did not show any significant differences based on smoking status, COPD severity, or the season in which the sample was collected (Supplementary Figures 1C–E). In stratified analysis conducted based on smoking status and sampling season, the SD group showed significantly higher alpha diversity compared to the AE group (Supplementary Figures 2C,D).

In the longitudinal comparison of AE samples, observed feature counts, Shannon’s index, and Fisher’s alpha index showed a decrease after 2 weeks (AE-1 vs. AE-2; 104.0 [74.0–136.0] vs. 92.0 [38.0–110.0], *p* = 0.011; 3.5 [3.0–3.9] vs. 3.1 [2.2–3.9], *p* = 0.023; and 13.2 [9.2–18.1] vs. 11.2 [4.2–14.0], *p* = 0.006, respectively, all by paired Wilcoxon test; Figures 1A–C). Antibiotic use during ECOPD did not result in a significant decrease in diversity (Supplementary Figure 1B). Additionally, AE-1 showed higher alpha diversity compared to AE-2 in stratified analysis by antibiotic use (Supplementary Figure 2B).

In the beta diversity analysis, un-weighted UniFrac showed a significant difference between the SD and AE-1 groups (*R*^2^ = 0.031, *p* = 0.041 by PERMANOVA; Figure 1D). However, no significant differences were observed in weighted UniFrac and Bray–Curtis dissimilarity between the SD and AE-1 groups (*R*^2^ = 0.024, *p* = 0.724 and *R*^2^ = 0.025, *p* = 0.841, respectively; Figures 1E,F). In the longitudinal comparison of AE samples, the centroid distance of Bray–Curtis dissimilarity was significantly higher in AE-2 compared to AE-1 (0.656 [0.644–0.674] vs. 0.669 [0.653–0.691], *p* = 0.006 by paired Wilcoxon test; Figures 1G,H). Bray–Curtis dissimilarity, unweighted UniFrac, and weighted UniFrac analyses for beta diversity also showed significant differences according to COPD stage (*R*^2^ = 0.029, *p* = 0.001 by PERMANOVA; *R*^2^ = 0.030, *p* = 0.001; *R*^2^ = 0.033, *p* = 0.006; Figures 1I–K, respectively).

### Distribution of airway microbiomes in patients with COPD

Supplementary Figures 3, 4 illustrate the overall distribution of the microbiome. The predominant phyla in AE-1 and SD were *Bacillota* (77.5% [57.1–82.7] and 59.2% [40.2–75.3], *p* = 0.107, respectively), *Pseudomonadota* (7.4% [4.7–27.4] and 26.8% [16.4–45.6], *p* = 0.101, respectively), and *Actinomycetota* (3.5% [2.2–7.3] and 6.4% [2.1–9.4], *p* = 0.322, respectively). *Fusobacteriota* was significantly reduced in the AE-1 compared to the SD (0.2% [0.1–1.0] vs. 1.2% [0.4–1.7], *p* = 0.016).

At the genus level, significant differences were observed for *Neisseria* (AE-1 vs. SD; 2.6% [0.1–7.4] vs. 17.1% [3.6–38.4], *p* = 0.022), *Fusobacterium* (0.2% [0.1–0.8] vs. 0.7% [0.2–1.1], *p* = 0.049), *Leptotrichia* (0.1% [0.0–0.2] vs. 0.3% [0.1–0.6], *p* = 0.038), *Bergeyella* (0.0% [0.0–0.0] vs. 0.1% [0.0–0.2], *p* < 0.001), *Peptostreptococcus* (0.0% [0.0–0.0] vs. 0.0% [0.0–0.2], *p* = 0.041), *Oribacterium* (0.0% [0.0–0.1] vs. 0.2% [0.0–0.5], *p* = 0.048). At the species level, significant differences were found in *Neisseria perflava* (0.6% [0.0–2.4] vs. 6.1% [1.6–37.9], *p* = 0.011) and *Haemophilus parainfluenzae* (0.8% [0.0–2.1] vs. 6.0% [4.4–7.9], *p* = 0.011).

Among these genera, the proportions of *Fusobacterium* (under 73 vs. 73 and older: 0.7% [0.2–1.4] vs. 0.2% [0.0–0.7], *p* = 0.031), *Leptotrichia* (0.2% [0.0–0.6] vs. 0.0% [0.0–0.2], *p* = 0.019), and *Oribacterium* (0.1% [0.0–0.5] vs. 0.0% [0.0–0.1], *p* = 0.012) were significantly higher in patients under 73 years compared to those aged 73 and older (Supplementary Figure 5A). Similarly, among COPD GOLD grades, the proportions of *Fusobacterium* (grades 1–2 vs. 3–4: 1.0% [0.2–1.7] vs. 0.2% [0.0–0.5], *p* = 0.001), *Bergeyella* (0.1% [0.0–0.1] vs. 0.0% [0.0–0.0], *p* < 0.001), *Peptostreptococcus* (0.0% [0.0–0.1] vs. 0.0% [0.0–0.0], *p* = 0.006), and *Oribacterium* (0.1% [0.0–0.4] vs. 0.0% [0.0–0.2], *p* = 0.046) were significantly higher in patients with GOLD grades 1–2 compared to those with grades 3–4 (Supplementary Figure 5B).

### Dynamics of airway microbiome in initiation of ECOPD

**Figure 2:**
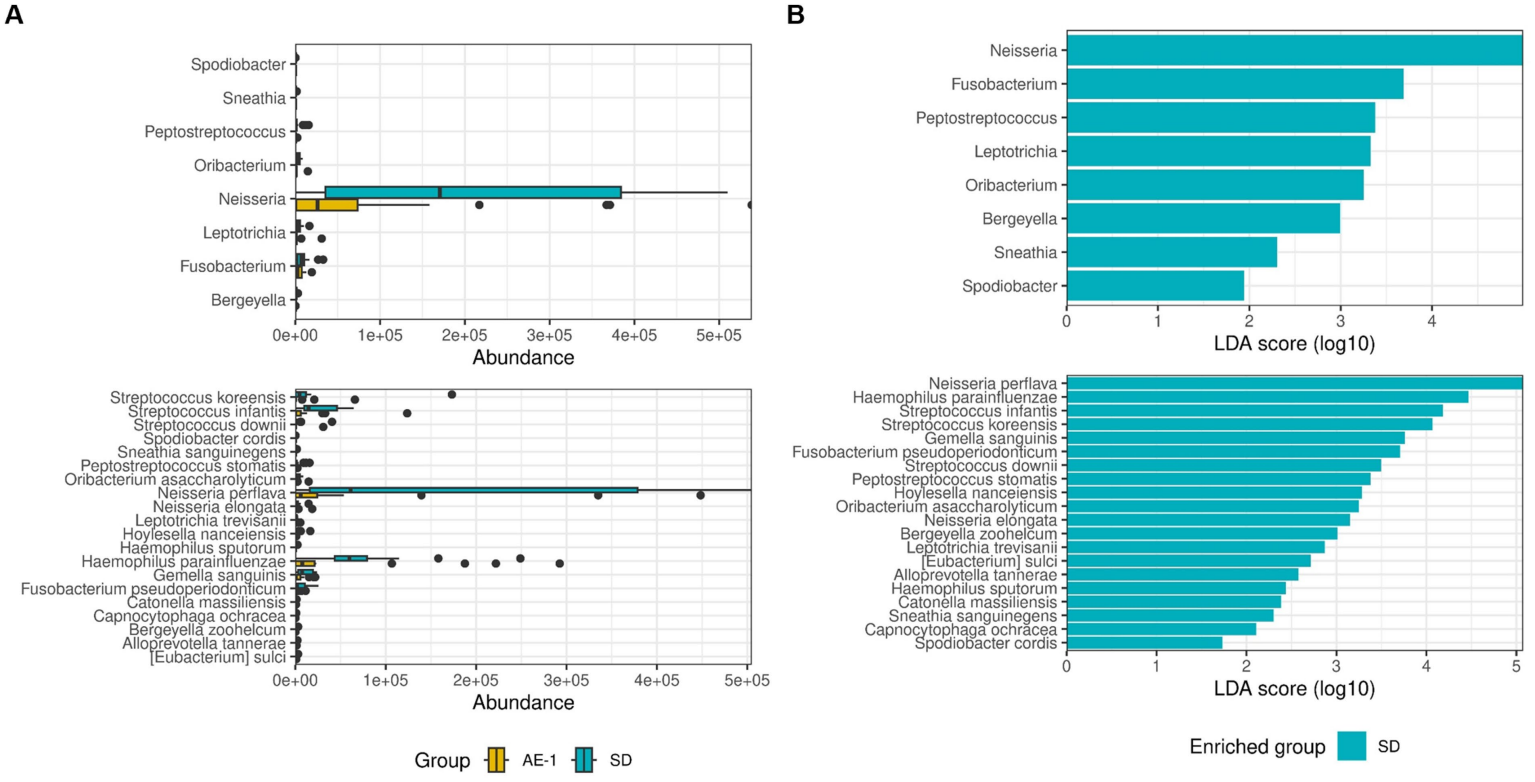
Comparison of the proportion of airway microbiome between groups. Panel **(A)** displays whisker-bar plots of genera and species with significantly increased abundance when comparing SD to AE-1. Panel **(B)** shows a bar plot from linear discriminant analysis (LDA) effect size (LEfSe), highlighting genera and species with significantly higher abundance in each group (SD and AE-1); no genera were found to be significantly increased in AE-1.

LEfSe analysis revealed that the SD group showed significantly higher abundances of *Neisseria* (LDA 4.996, adjusted P [adj.P] = 0.021), *Fusobacterium* (LDA 3.688, adj.*p* = 0.047), and *Peptostreptococcus* (LDA 3.379, adj.*p* = 0.039) at the genus level compared to the AE-1 group. At the species level, *N. perflava* (LDA 5.074, adj.*p* = 0.010) and *H. parainfluenzae* (LDA 4.467, adj.*p* = 0.011) were significantly more abundant in the SD group compared to the AE-1 group (Figure 2; Supplementary Figure 6). No genera or species in the AE-1 group showed significantly higher abundance compared to the SD group (Figure 2).

**Figure 3:**
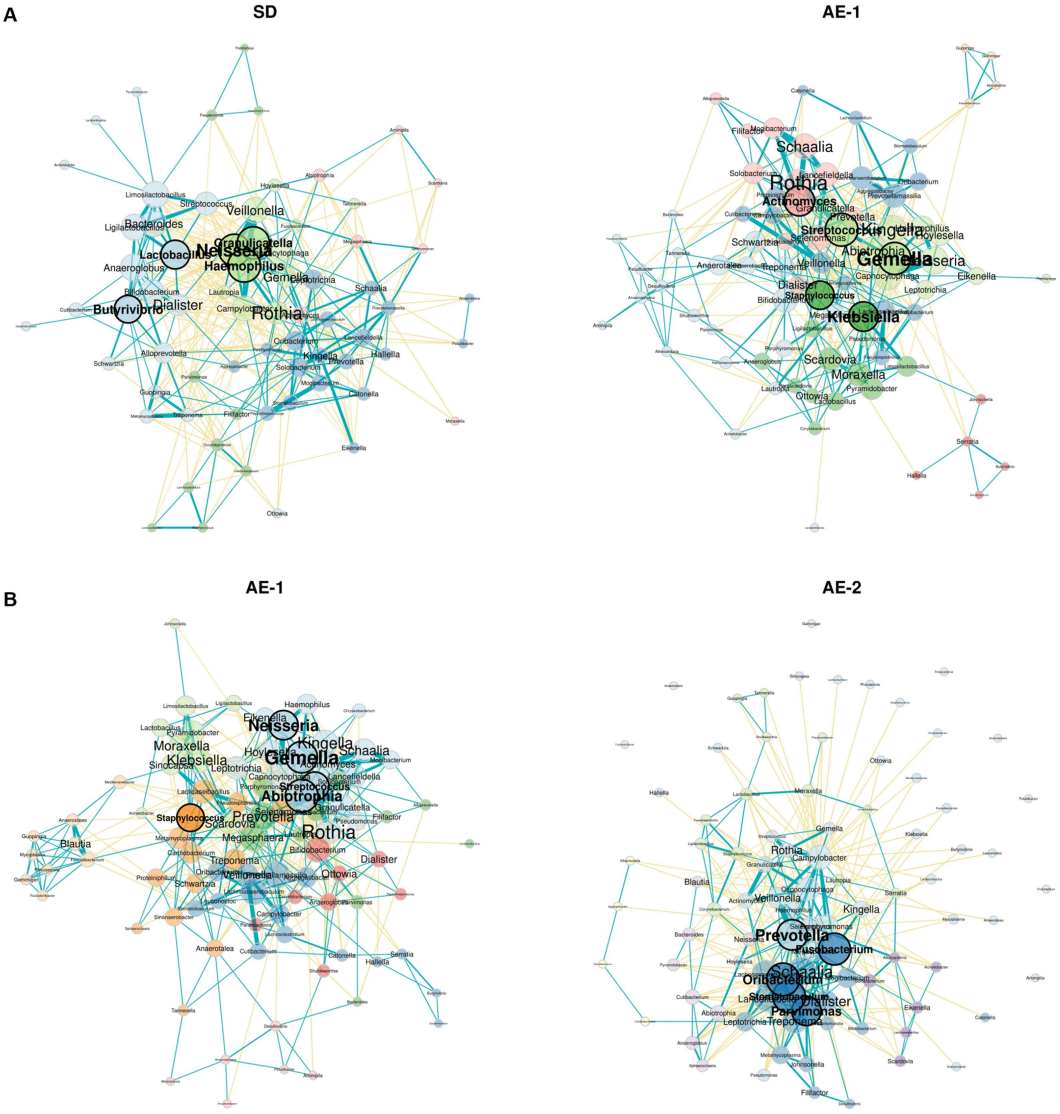
Network analysis of airway microbiome from onset to recovery of COPD exacerbation. Panels **(A,B)** display network analyses of genera for each sample, comparing SD vs. AE-1 and AE-1 vs. AE-2, respectively. Node color represents phylum classification, and node size is proportional to eigenvector centrality. Turquoise lines indicate significant positive correlations between genera, while yellow lines represent significant negative correlations.

Network analysis revealed that the SD group had a more complex and stronger microbial network than the AE-1 group (0.155 vs. 0.108 for edge density and 0.034 vs. 0.023 for natural connectivity, respectively; Figure 3A). Hub genera identified based on empirical quantiles of centralities included *Haemophilus*, *Granulicatella*, *Neisseria*, *Lactobacillus*, and *Butyrivibrio* in the SD group and *Streptococcus*, *Gemella*, *Actinomyces*, *Klebsiella*, and *Staphylococcus* in the AE-1 group.

### Dynamics of airway microbiomes in the recovery from exacerbation of COPD

**Figure 4:**
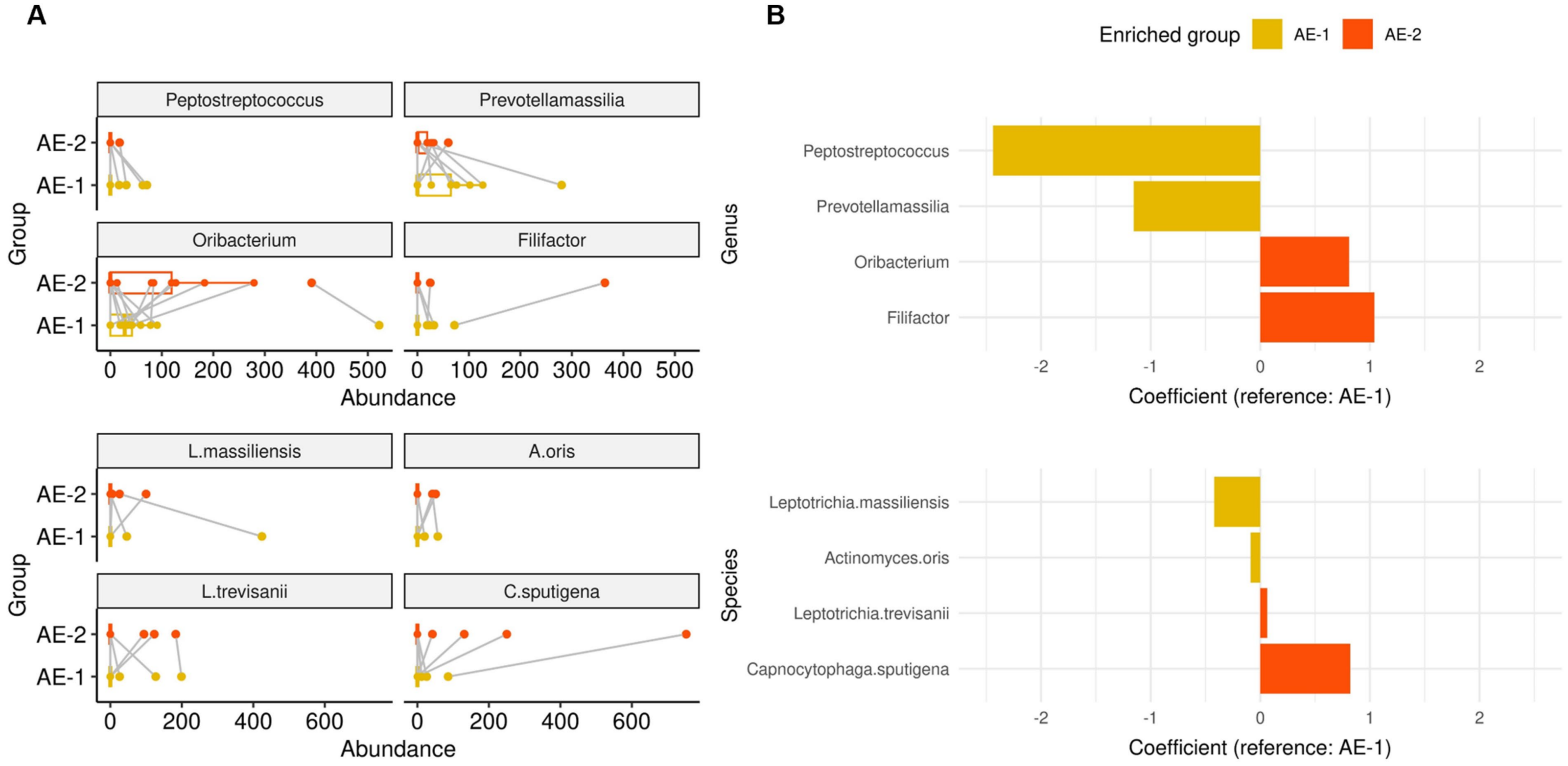
Dynamics of airway microbiome in onset of COPD exacerbation. Panels **(A,B)** displays whisker-bar and bar plot of genera and species with significantly increased abundance when comparing AE-1 to AE-2, analyzed using a mixed-effects linear model for paired samples with “subject” set as a random effect for within-patient analysis.

During the recovery process from ECOPD, the AE-2 group showed a significant increase in the abundance of the genera *Filifactor* (coefficient 1.042, *p* < 0.001) and *Oribacterium* (coefficient 0.811, *p* = 0.008) compared to the AE-1 group, as analyzed by a mixed-effects linear model for longitudinal analysis (Figure 4). In contrast, *Prevotellamassilia* (coefficient − 1.154, *p* = 0.003) and *Peptostreptococcus* (coefficient − 2.438, *p* = 0.005) showed a significant decrease in AE-2. At the species level, the AE-2 group showed a significant increase in the abundance of the species *C. sputigena* (coefficient 0.821, *p* < 0.001) and *L. trevisanii* (coefficient 0.063, *p* < 0.001) compared to the AE-1 group. In contrast, *A. oris* (coefficient − 0.087, *p* < 0.001) and *L. massiliensis* (coefficient − 0.422, *p* < 0.001) showed a significant decrease in AE-2.

Network analysis revealed that AE-2 had a more complex microbial network than AE-1 (natural connectivity: 0.031 vs. 0.022; Figure 3B). The hub genera identified in the AE-1 group included *Abiotrophia*, *Gemella*, *Neisseria*, *Streptococcus*, and *Staphylococcus*. In contrast, the AE-2 group featured hubs such as *Oribacterium*, *Stomatobaculum*, *Fusobacterium*, *Prevotella*, and *Parvimonas*.

### The correlation between airway microbiome and clinical parameters

**Figure 5:**
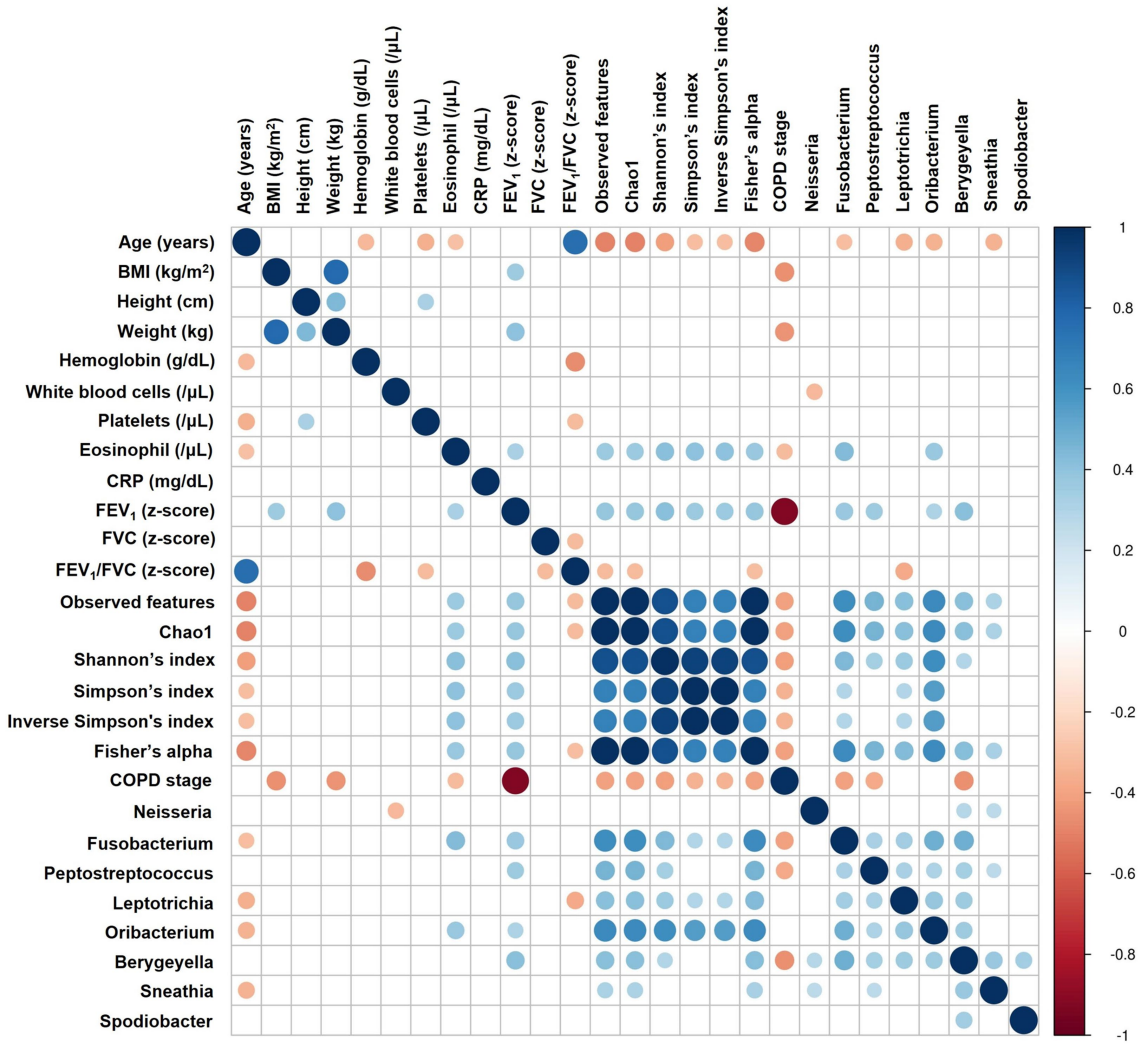
Correlation analysis between airway microbiome and clinical parameters. The heatmap displays Spearman correlation (rho) between variables, with significant positive correlations shown in blue, significant negative correlations in red, and statistically non-significant correlations (*p* ≥ 0.05) left blank. The size of each circle is inversely proportional to the *p*-value, with larger circles indicating more statistically significant correlations. BMI, Body Mass Index; CRP, C-reactive protein; FEV_1_, Forced Expiratory Volume in 1 s; FVC, Forced Vital Capacity; COPD, Chronic Obstructive Pulmonary Disease.

Spearman correlation analysis revealed that alpha diversity was significantly negatively correlated with age (Shannon’s index: rho = −0.413, *p* = 0.002) and COPD stage (rho = −0.414, *p* = 0.002; Figure 5A; Supplementary Table 1). Conversely, alpha diversity positively correlated with serum eosinophil levels (rho = 0.413, *p* = 0.003) and the z-score of FEV_1_ (rho = 0.416, *p* = 0.002). *Fusobacterium*, *Peptostreptococcus*, *Oribacterium*, and *Bergeyella* were positively correlated with FEV_1_ and alpha diversity indices (Figure 5; Supplementary Table 2). Supplementary Figure 7 and Supplementary Table 3 describe additional correlations with other species.

## Discussion

This study demonstrated that the distribution and complexity of the lung microbiome are associated with ECOPD. Patients with ECOPD showed an observed decrease in certain bacteria typically present in the normal airway flora compared to stable COPD patients. This suggests that acute exacerbations of COPD may not primarily result from an increase in pathogenic bacteria but rather from changes in the colonization of normal flora.

This finding was supported by microbial network analysis, which highlighted the diversity and interactions of bacterial strains constituting the normal airway microbial flora as crucial factors in the onset and recovery from ECOPD.

During ECOPD, a significant decrease in the *Neisseria* genus was observed, specifically a reduction in *N. perflava*. The genus *Neisseria* includes two clinically significant pathogenic species: *N. gonorrhoeae* and *N. meningitidis*. In contrast, *N. perflava* is a part of the normal flora and is generally considered non-pathogenic (Seifert, 2019). *Neisseria* is commonly found in patients with COPD (Karakasidis et al., 2023; Wang et al., 2020; Zakharkina et al., 2013; Pragman et al., 2018). Although research on the impact of *Neisseria* on ECOPD is limited, several possibilities that could influence ECOPD have been suggested. Morris et al. (2013) reported a reduction in *Neisseria* within the microbiome of smokers, one of the critical risk factors for ECOPD. Additionally, Kim et al. (2019) reported that commensal *Neisseria* species can induce cell death of pathogens. Our study provides indirect evidence that commensal *Neisseria* may reduce ECOPD. Notably, *Neisseria* was not only numerically significant, but also served as a central hub in the microbial network of patients with SD, suggesting that it may play a crucial role in regulating normal flora. However, further studies are required to confirm this hypothesis.

In this study, *Haemophilus parainfluenzae* also showed a significant negative association with ECOPD. *H. parainfluenzae* is one of the pathogens that cause relatively rare opportunistic infections (Hill et al., 2000). As part of the normal flora, *Haemophilus* plays a critical role in preventing the colonization of potential pathogens and is essential for the body’s defense mechanisms (Dickson et al., 2013; Kosikowska et al., 2016). Wang et al. (2020) reported that *H. parainfluenzae* plays an anti-inflammatory role in patients with COPD. This study revealed that *H. parainfluenzae* might play a role in preventing ECOPD, both quantitatively and functionally.

The role of specific species is important in ECOPD; however, the overall diversity and robustness of the microbial network systems are also crucial. Alpha diversity of samples was significantly higher in patients with SD than in those with ECOPD.

Recent studies have identified decreased microbiome diversity as a significant risk factor for ECOPD. Pragman et al. found that the COPD frequent exacerbator phenotype was associated with decreased sputum microbiome alpha diversity and increased beta diversity (Pragman et al., 2024). In our study, alpha diversity decreased with increasing age and COPD severity. A significant correlation with FEV_1_ was also observed, with higher FEV_1_ values associated with increased alpha diversity. This suggests that diversity is closely related not only to ECOPD but also to symptoms and prognosis. Previous studies have reported that the diversity of the airway microbiome decreases with age (Pragman et al., 2024; Mayhew et al., 2018; Millares et al., 2019). This decline is associated with increased susceptibility to lung infections and reduced lung function. In our study, we adjusted forced vital capacity (FVC), FEV_1_, and FEV_1_/FVC for age, height, sex, and race, converting them to z-scores using the GLI-2012 reference. We found a significant negative correlation in only FEV_1_, suggesting that the limitation of expiration significantly affected microbiome diversity.

During ECOPD, the disrupted balance and decreased diversity of the airway microbiome did not recover in the short term, even after the exacerbation period ended. Specifically, the alpha diversity showed a significant decrease when comparing ECOPD onset and 2 weeks after treatment. This decrease in diversity may be attributed to the use of antibiotics and steroids during the exacerbation period. The results indicate that the SD group has higher microbial diversity and a more stable community, whereas the AE group experiences a decline in diversity and shifts in community composition over time. This suggests that ECOPD condition may gradually reduce microbial community stability.

Additionally, genera such as *Neisseria*, *Haemophilus*, *Lactobacillus*, and *Butyrivibrio*, which play crucial roles in the microbial network of patients with SD, did not show significant changes over the 2 weeks. Therefore, further research is needed to identify the causes of microbial diversity disruption and develop treatments that help restore this diversity, which is crucial for preventing the occurrence and recurrence of ECOPD.

This study had several strengths. Through a prospective study design, we obtained clinical information from patients and secured samples 2 weeks after ECOPD. This allowed us to analyze the microbiome not only during exacerbations but also during recovery. Additionally, patients with a history of antibiotic use within the previous month were excluded to minimize the impact of recent antibiotic use on the results.

However, this study also had several limitations. First, the sample size was small, and as a single-center study, there is a possibility that statistical significance may not have been achieved. Especially, four patients experienced follow-up loss, resulting in the inability to obtain four AE-2 samples. Consequently, these patients were excluded from the analysis comparing AE-1 and AE-2 samples, which may have impacted the results. The small sample size limits the interpretation of longitudinal analysis, and network analysis was conducted using a cross-sectional approach. Future studies should address these limitations and refine longitudinal techniques to better assess the impact of ECOPD on the microbiome. Second, the patient enrollment process was slow due to a recruitment period of approximately 4 years. The onset of the COVID-19 pandemic during the study period necessitated a temporary halt in enrollment, which may have affected the study outcomes. Lastly, unadjusted confounding factors may have influenced the results. These factors include antibiotics, steroids, age, COPD stage, and smoking. Although stratified or multivariate analysis would be ideal for adjustment, the small sample size in this study limited our ability to perform these analyses.

## Conclusion

Exacerbations in patients with COPD are associated with increases and decreases in specific strains of normal flora. However, this study suggests that beyond these changes, maintaining the diversity of flora and the regulation and balance within the microbial network may be more crucial in ECOPD. Further research on the specific biological pathways and their regulation is required.

## Data Availability

The datasets presented in this study can be found in online repositories. The names of the repository/repositories and accession number(s) can be found at: https://www.ncbi.nlm.nih.gov/, PRJNA1162680. The analysis was executed using R version 4.3.2 (2023-10-31). All code files are available for access on GitHub at https://github.com/0717cyj/COPD_microbiome.

## Footnotes

^1^ https://www.pacb.com/

^2^ https://www.R-project.org/
