# Supplementary material for: Dynamic microbial changes in exacerbation of chronic obstructive pulmonary disease
Source: Front Microbiol. 2024 Dec 6;15:1507090. doi: 10.3389/fmicb.2024.1507090 (PMC11659282; doi:10.3389/fmicb.2024.1507090)
Supplement: Supplementary file 1 [file Data_Sheet_1.PDF]

Supplementary Figure 1. Diversity of Airway Microbiome in COPD Patients by Age, Antibiotic Use, Smoking Status, Season of Sampling, and COPD Grade

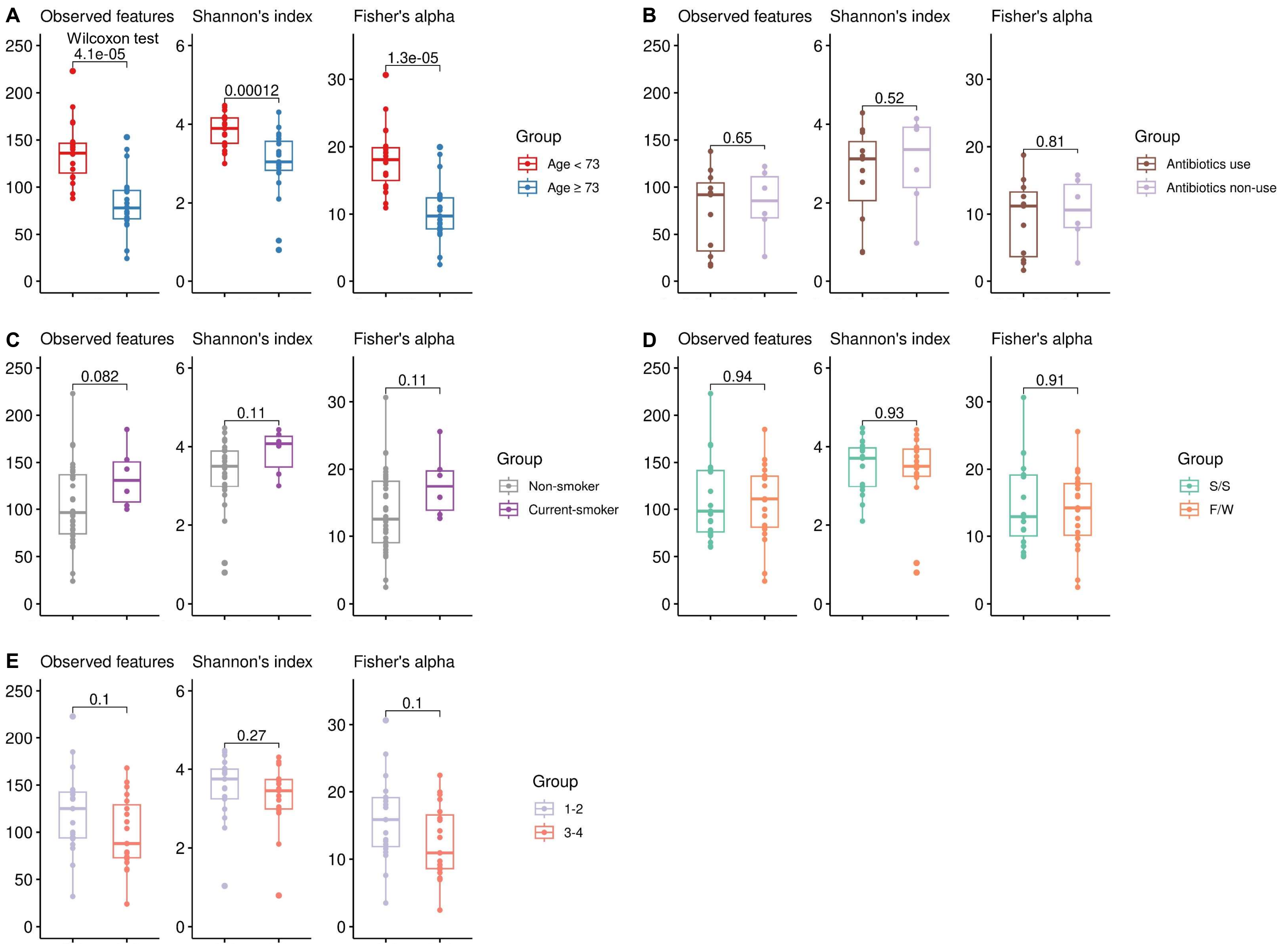

Panels A–E display whisker bar plots comparing observed features, Shannon's index, and Fisher's alpha index across age, antibiotic use, smoking status, season, and GOLD group, respectively. All P-values were calculated using the Wilcoxon test, with each point representing an individual sample.

Abbreviations: COPD, Chronic Obstructive Pulmonary Disease; S/S, Spring/Summer; F/W, Fall/Winter.

Supplementary Figure 2. Diversity of Airway Microbiome in COPD Patients stratified by Age, Antibiotics Use, Smoking Status, Season of Sampling, and COPD Grade

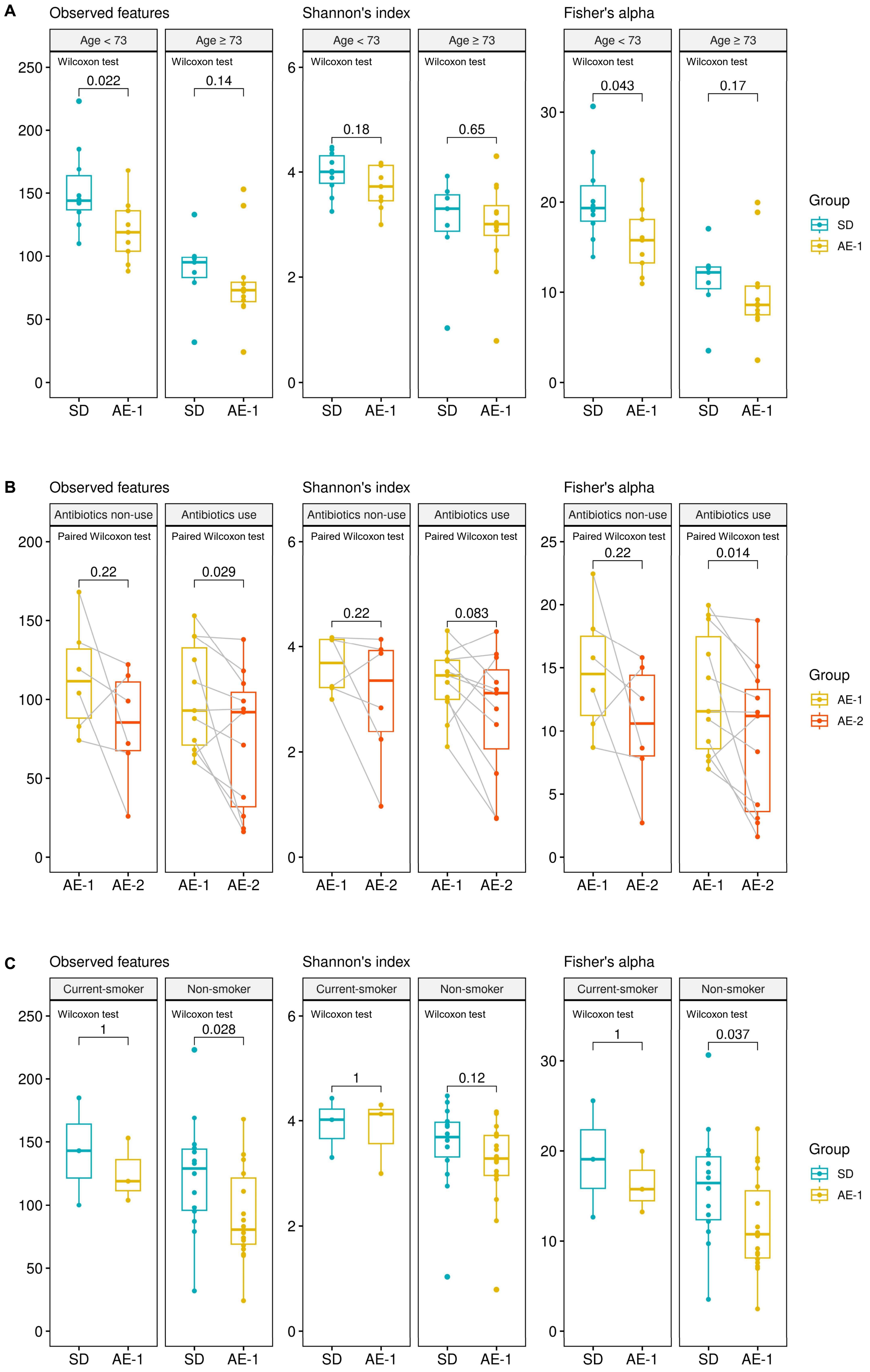

Supplementary Figure 2. (continued)

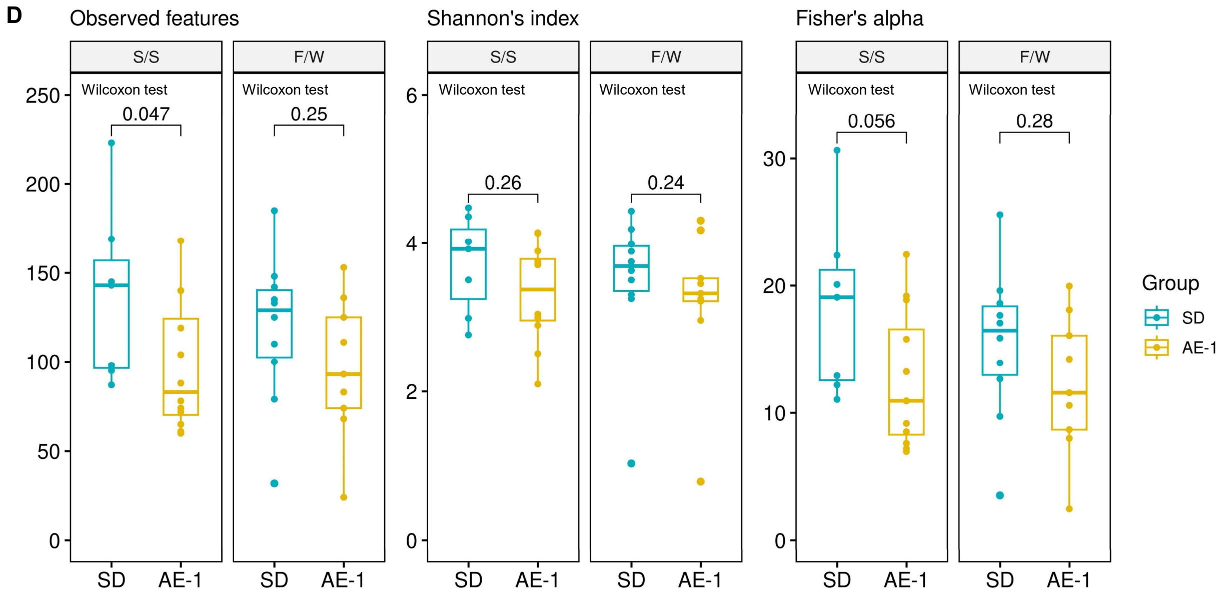

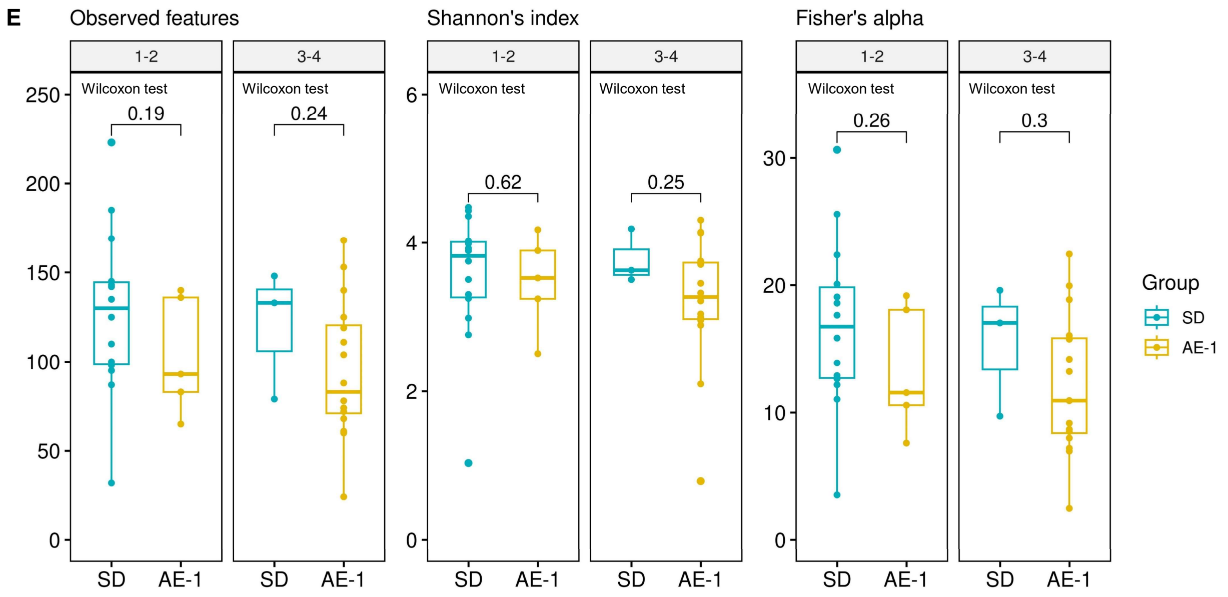

Panels A–E display whisker bar plots comparing observed features, Shannon's index, and Fisher's alpha index between SD vs. AE-1 or AE-1 vs. AE-2, stratified by age, antibiotic use, smoking status, season, and GOLD group, respectively. P-values were calculated using the Wilcoxon test for comparisons between SD and AE-1, and the paired Wilcoxon test for comparisons between AE-1 and AE-2. Each point represents an individual sample.

Abbreviations: COPD, Chronic Obstructive Pulmonary Disease; S/S, Spring/Summer; F/W, Fall/Winter.

Supplementary Figure 3. Distribution of airway microbiomes in patients with COPD

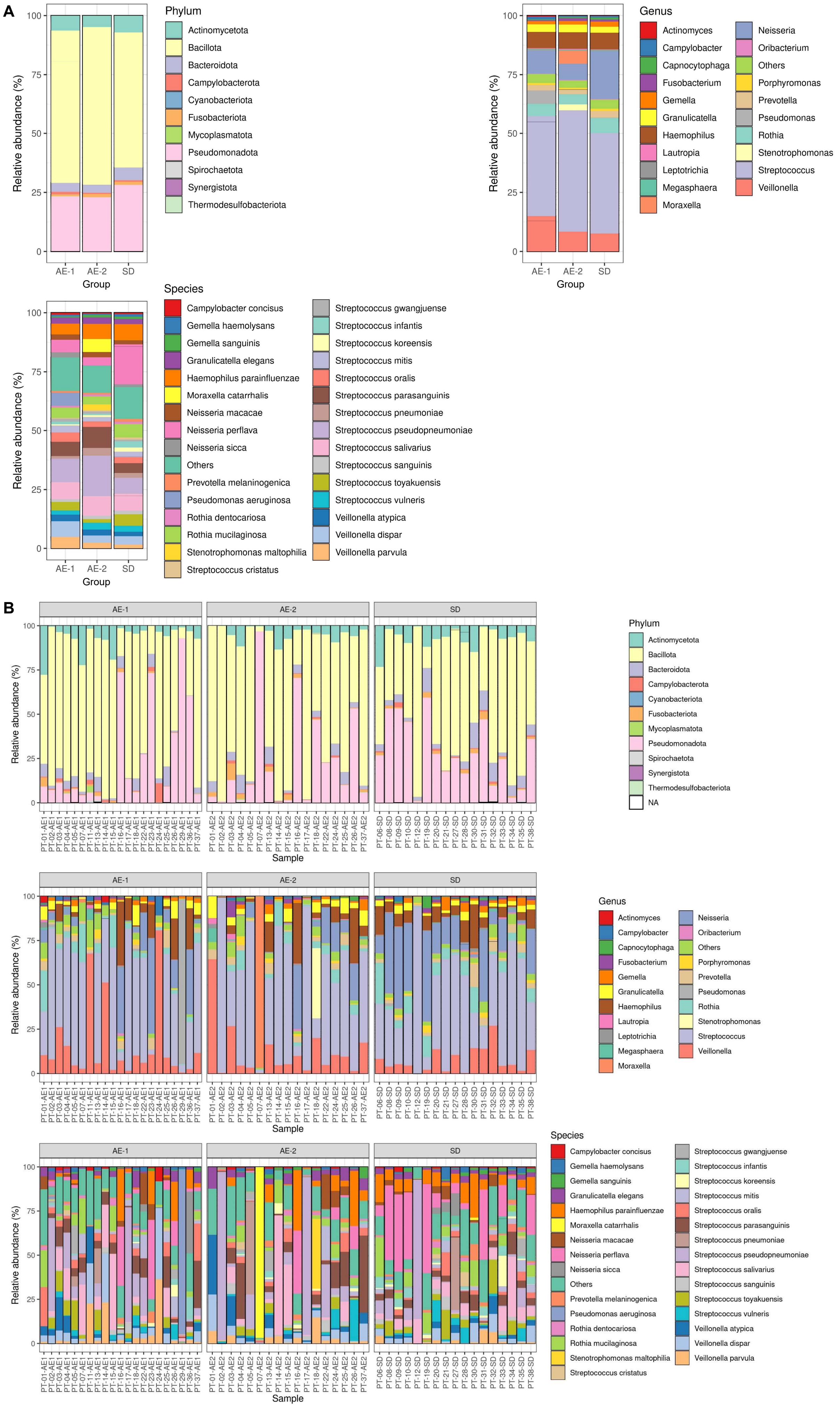

Panel A shows the relative abundance of phyla, genera, and species between the stable disease (SD) and exacerbation groups (AE-1 and AE-2), while Panel B displays the abundance within each sample as a bar plot.

Supplementary Figure 4. Proportion of airway microbiomes in patients with COPD

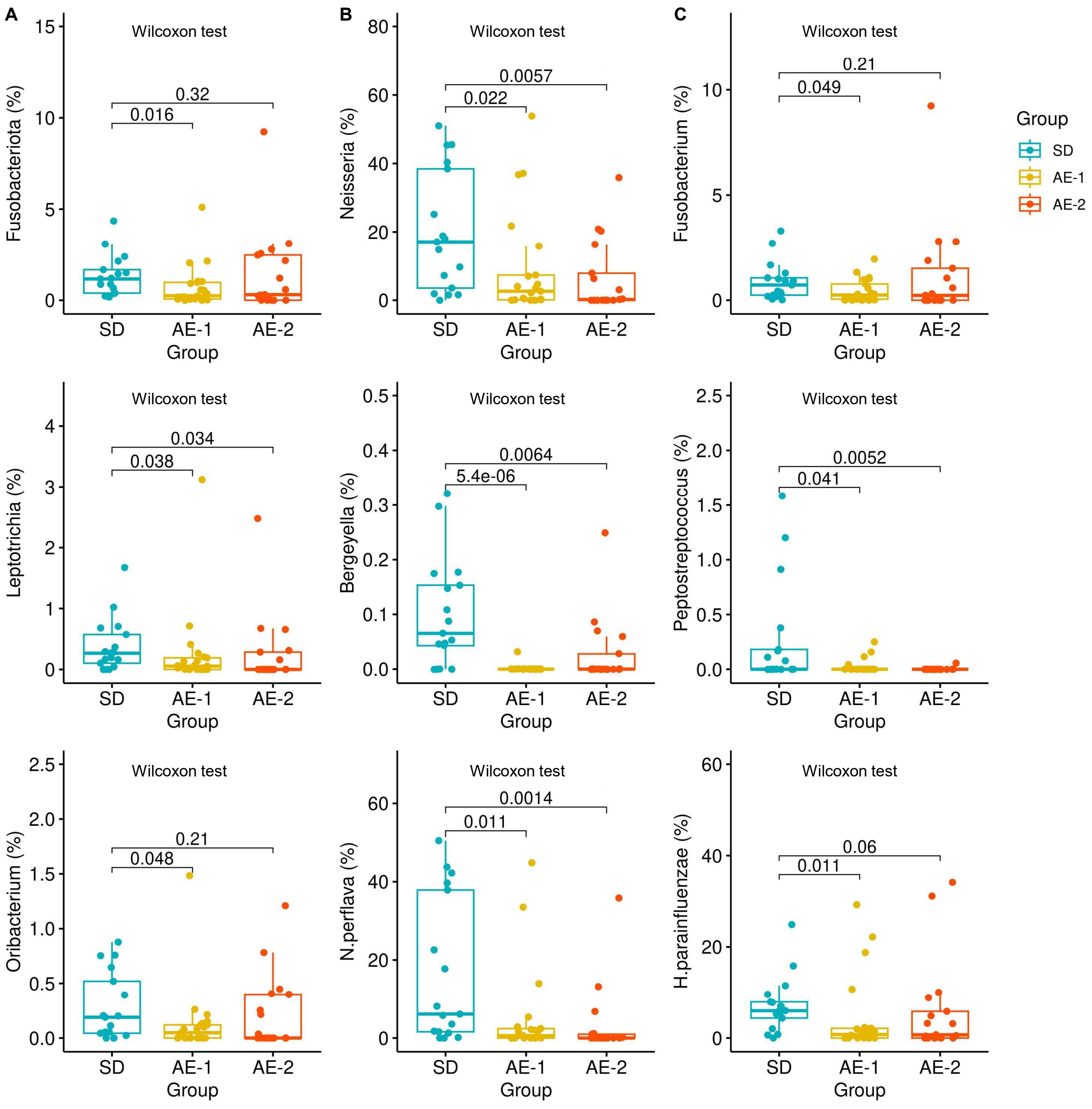

Panels A–E display whisker bar plots comparing the proportion of airway microbiomes in COPD patients between SD vs. AE-1 or SD vs. AE-2. The y-axis represents the relative proportion of each genus or species. P-values were calculated using the Wilcoxon test for comparisons, with each point representing an individual sample.

Supplementary Figure 5. Distribution of airway microbiomes in patients with COPD by age and GOLD grade

A

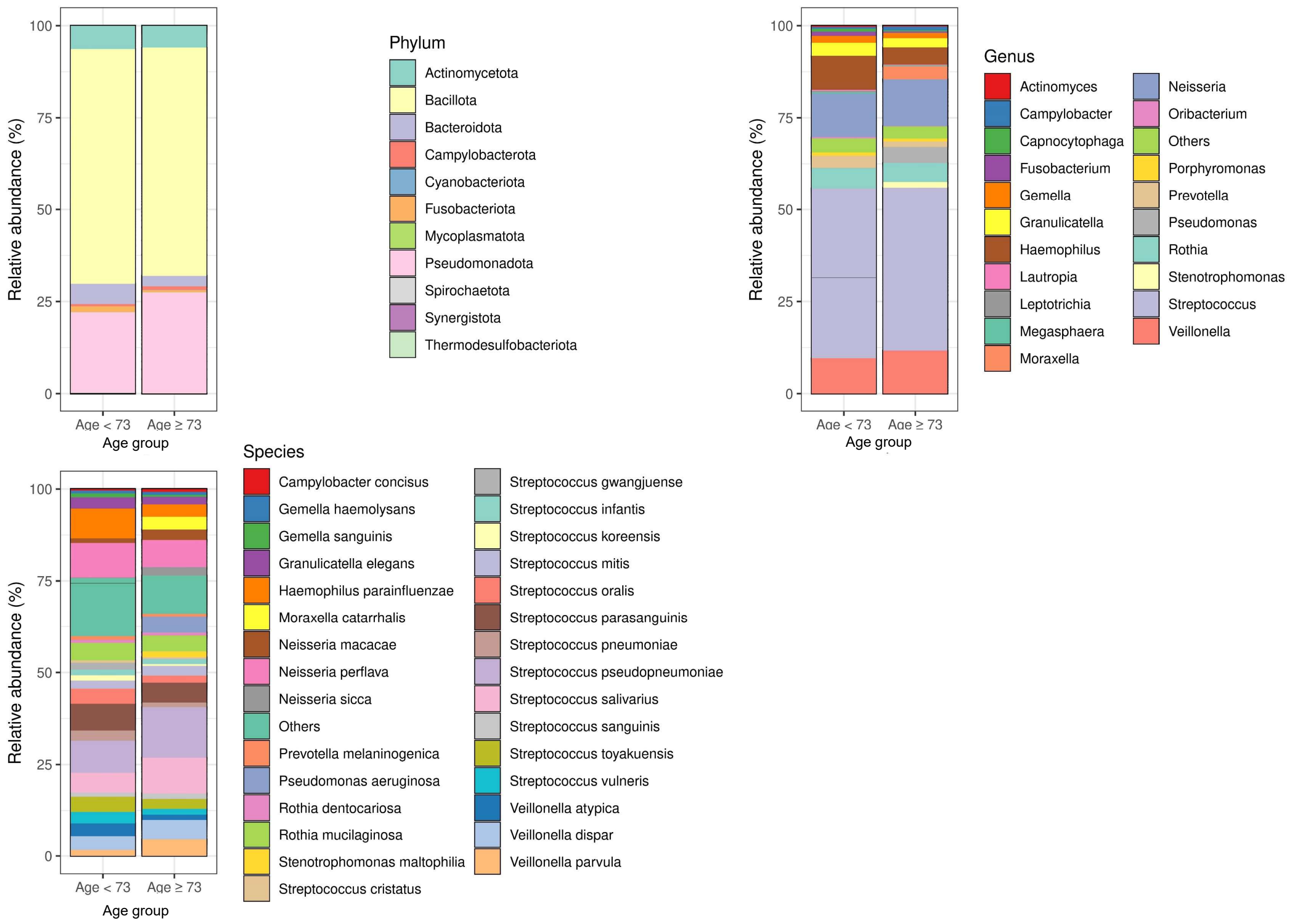

B

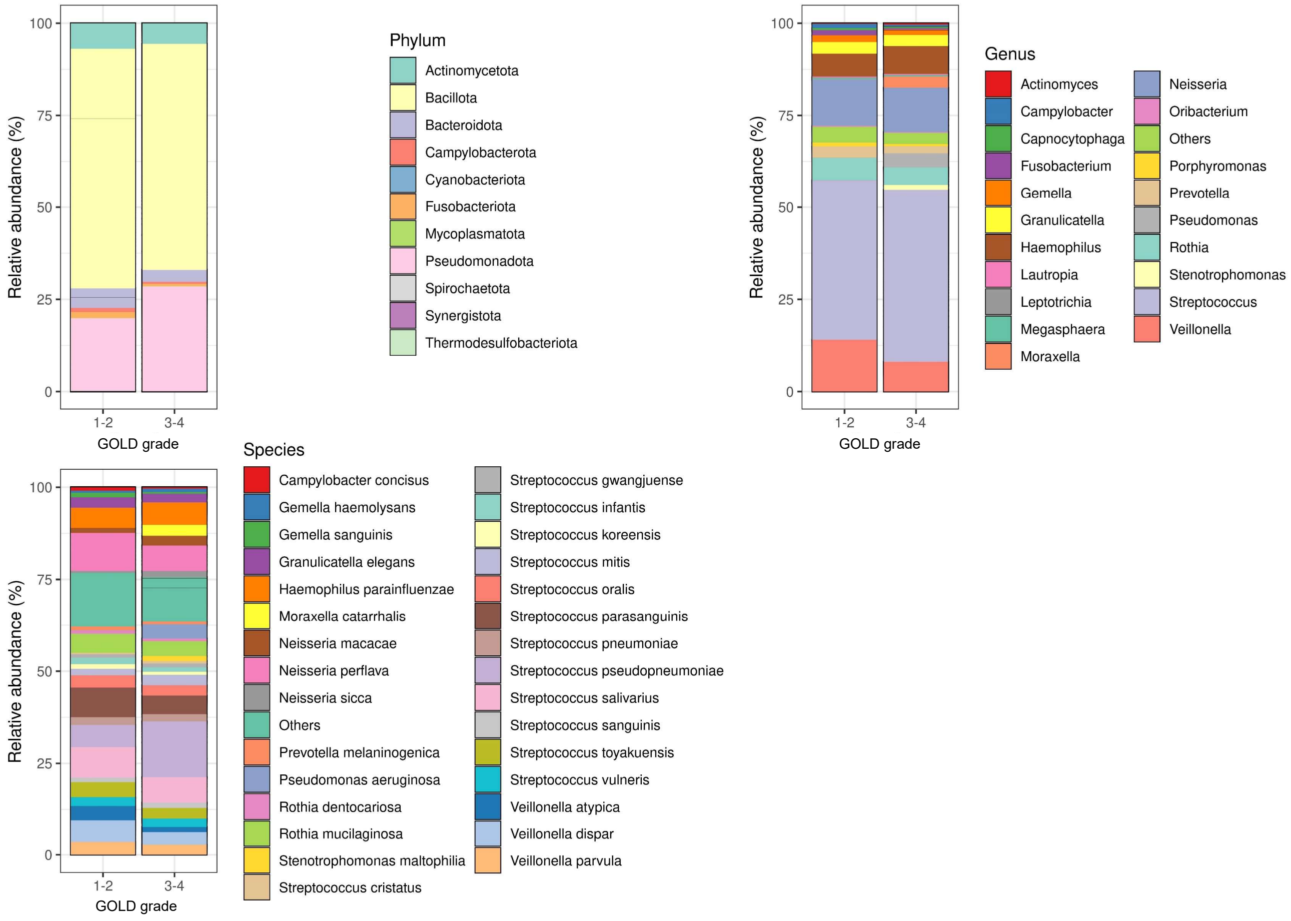

Panels A and B are bar plots comparing the relative abundance of phyla, genera, and species between age groups under 73 vs. 73 and older, and COPD GOLD grades 1–2 vs. 3–4, respectively.

Supplementary Figure 6. Dynamics of Airway Microbiome in Onset of COPD Exacerbation

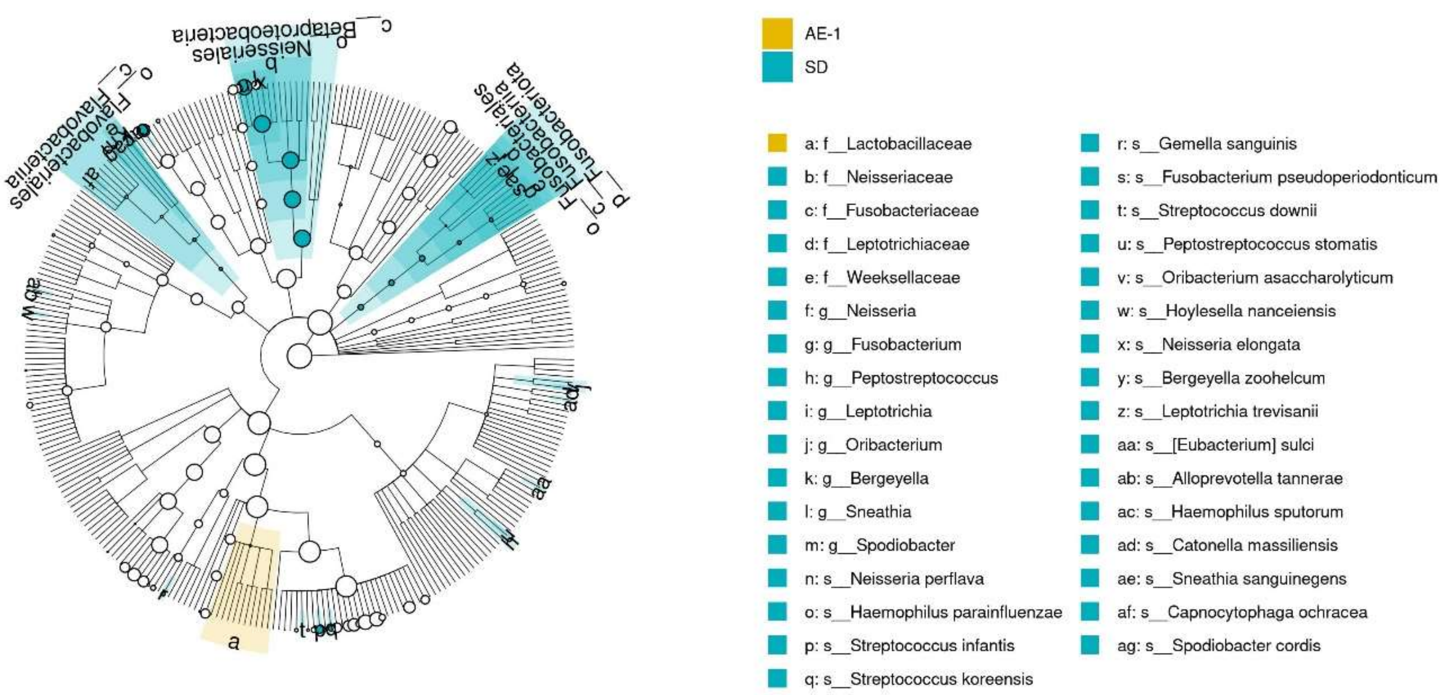

The cladogram showing the phylogenetic relationships of taxa, with highlights indicating significant differences in abundance between the AE-1 (yellow) and SD (blue) groups.

Supplementary Figure 7. Correlation analysis between airway microbiome and clinical parameters

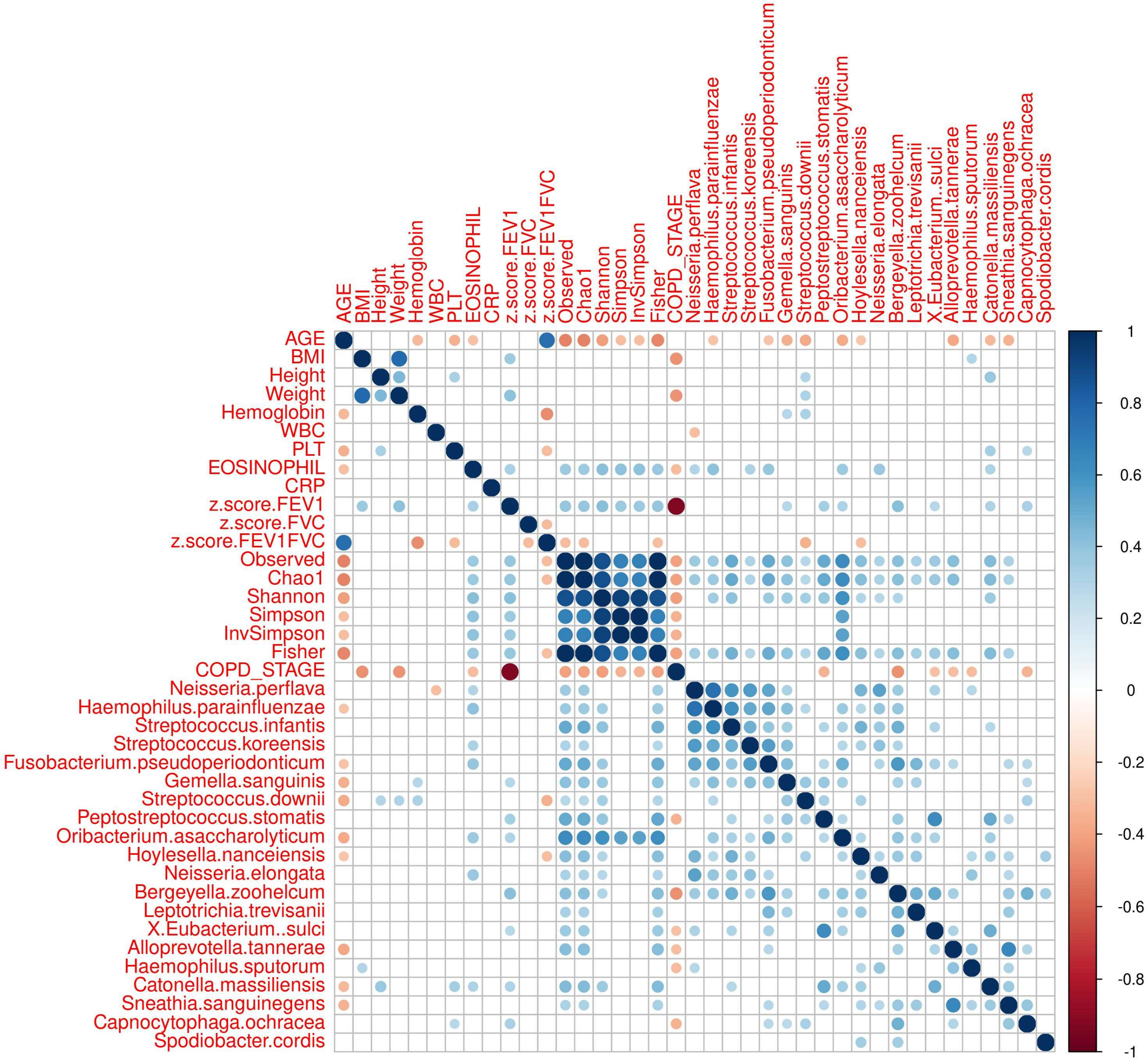

The heatmap displays Spearman correlation (rho) between variables, with significant positive correlations shown in blue, significant negative correlations in red, and statistically non-significant correlations (P ≥ 0.05) left blank. The size of each circle is inversely proportional to the P-value, with larger circles indicating more statistically significant correlations.

Abbreviations: BMI, Body Mass Index; CRP, C-reactive protein; FEV1, Forced Expiratory Volume in 1 second; FVC, Forced Vital Capacity; COPD, Chronic Obstructive Pulmonary Disease
